# NadA3 Structures Reveal Undecad Coiled Coils and LOX1 Binding Regions Competed by Meningococcus B Vaccine-Elicited Human Antibodies

**DOI:** 10.1128/mBio.01914-18

**Published:** 2018-10-16

**Authors:** Alessia Liguori, Lucia Dello Iacono, Giulietta Maruggi, Barbara Benucci, Marcello Merola, Paola Lo Surdo, Jacinto López-Sagaseta, Mariagrazia Pizza, Enrico Malito, Matthew J. Bottomley

**Affiliations:** a GSK, Siena, Italy; b Department of Biology, University of Naples Federico II, Naples, Italy; Pasteur Institute; École Polytechnique Fédérale de Lausanne; University of Bristol

**Keywords:** LOX-1 receptor, neisserial adhesin A, monoclonal antibody epitopes, three-dimensional structure, vaccine antigen

## Abstract

Neisseria meningitidis serogroup B (MenB) is a major cause of sepsis and invasive meningococcal disease. A multicomponent vaccine, 4CMenB, is approved for protection against MenB. Neisserial adhesin A (NadA) is one of the main vaccine antigens, acts in host cell adhesion, and may influence colonization and invasion. Six major genetic variants of NadA exist and can be classified into immunologically distinct groups I and II. Knowledge of the crystal structure of the 4CMenB vaccine component NadA3 (group I) would improve understanding of its immunogenicity, folding, and functional properties and might aid antigen design. Here, X-ray crystallography, biochemical, and cellular studies were used to deeply characterize NadA3. The NadA3 crystal structure is reported; it revealed two unexpected regions of undecad coiled-coil motifs and other conformational differences from NadA5 (group II) not predicted by previous analyses. Structure-guided engineering was performed to increase NadA3 thermostability, and a second crystal structure confirmed the improved packing. Functional NadA3 residues mediating interactions with human receptor LOX-1 were identified. Also, for two protective vaccine-elicited human monoclonal antibodies (5D11, 12H11), we mapped key NadA3 epitopes. These vaccine-elicited human MAbs competed binding of NadA3 to LOX-1, suggesting their potential to inhibit host-pathogen colonizing interactions. The data presented provide a significant advance in the understanding of the structure, immunogenicity and function of NadA, one of the main antigens of the multicomponent meningococcus B vaccine.

## Abstract (precis)

The bacterial microbe Neisseria meningitidis serogroup B (MenB) is a major cause of devastating meningococcal disease. An approved multicomponent vaccine, 4CMenB, protects against MenB. Neisserial adhesin A (NadA) is a key vaccine antigen and acts in host cell-pathogen interactions. We investigated the 4CMenB vaccine component NadA3 in order to improve the understanding of its immunogenicity, structure, and function and to aid antigen design. We report crystal structures of NadA3, revealing unexpected structural motifs, and other conformational differences from the NadA5 orthologue studied previously. We performed structure-based antigen design to engineer increased NadA3 thermostability. Functional NadA3 residues mediating interactions with the human receptor LOX-1 and vaccine-elicited human antibodies were identified. These antibodies competed binding of NadA3 to LOX-1, suggesting their potential to inhibit host-pathogen colonizing interactions. Our data provide a significant advance in the overall understanding of the 4CMenB vaccine antigen NadA.

## INTRODUCTION

Neisseria meningitidis is a life-threatening bacterium that causes severe sepsis and meningococcal meningitis, often with rapid progression. Mortality associated with invasive meningococcal disease (IMD) can reach 10% to 20%, and survivors frequently suffer devastating long-term sequelae (1, 2). Estimates of global disease incidence reported approximately 500,000 cases and 50,000 deaths annually (3). Six meningococcal serogroups (A, B, C, W, Y, and X) are mainly responsible for IMD (4), and the serogroup B meningococcus (MenB) is now responsible for the majority of IMD in developed countries (5, 6).

The reverse-vaccinology approach was developed to generate a recombinant-protein-based vaccine to protect against MenB (7–11). The vaccine (termed 4CMenB [Bexsero]) has been licensed for use in all age groups from 2 months of age (5). Importantly, following nationwide introduction for infants in the United Kingdom in 2015, the 4CMenB vaccine approximately halved the number of cases of meningitis and septicemia caused by MenB infection in eligible infants (12, 13). In addition to containing an outer membrane vesicle (OMV) component, the 4CMenB vaccine contains three main recombinant meningococcal protein antigens, one of which is neisserial adhesin A (NadA) (11, 14, 15), the focus of this study.

The *nadA* gene is present in ∼30% of pathogenic meningococcal isolates and is often associated with hypervirulent MenB lineages (16). The expression level of *nadA* can vary over 100-fold, predominantly dependent on its transcriptional regulator, NadR, which responds to niche-specific signals (17, 18). Bactericidal titers correlate with NadA expression levels (19). NadA induces high levels of bactericidal antibodies in humans in all age groups (20), highlighting the key role of this antigen in vaccine-induced protection.

Two genetically and immunologically distinct groups of NadA exist, and they exhibit sequence identities of 45% to 50%. Group I members share ∼95% sequence identity and include the three most common variants, NadA1, NadA2, and NadA3, the last being the 4CMenB vaccine variant. Group II includes the rarer variants (NadA4, NadA5, and NadA6), which share ∼90% sequence identity. Generally, group I is associated with disease-causing strains, whereas group II is associated with carriage strains and with the sequence type 213 (ST213) complex. Cross-protection is induced by strains carrying NadA belonging to the same group, but not across groups.

NadA belongs to the OCA (oligomeric coiled-coil adhesin) family, a subgroup of the trimeric autotransporter adhesins (TAA), which generally mediate adhesion of Gram-negative bacteria to target cells or to the extracellular matrix and invasion. TAAs are virulence-promoting agents and share modular organizations, with an N-terminal domain involved in host recognition and a C-terminal translocation unit/membrane anchor domain (21). NadA3 promotes adhesion to and invasion of epithelial cells through its N-terminal region (22, 23). Internalization into human epithelial cells is mediated by an ARF6-regulated recycling pathway, as described for the major histocompatibility complex class I (MHC I) pathway and is dependent on Rab11 and Hsp90 (24, 25). Similarly, recombinant NadA was reported to stimulate human monocytes by binding Hsp90 (26, 27). It was recently demonstrated that the NadA3 N-terminal region binds specifically to the endothelial cell receptor LOX-1 (low-density oxidized lipoprotein lectin-like receptor 1) (28). The multiple interactions of NadA with a variety of host cells and receptors are likely to enable multiple host recognition mechanisms, each potentially contributing to meningococcal pathogenicity.

Despite its discovery over 15 years ago (9, 15), the three-dimensional (3D) structure of NadA3 has been elusive and remains enigmatic. While sequence-based predictions suggested a mixed tertiary structure of interrupted dimeric and trimeric coiled coils in NadA3 (23, 29), the first crystal structure of NadA5 revealed exclusively trimeric coiled-coil elements and a small globular head domain, comprised of a central trimeric coiled coil flanked by beta-hairpin wing motifs (30). The NadA5 crystal structure included only residues 24 to 137, and no structural information was available for the C-terminal region, including the predicted coiled coil and the highly conserved membrane anchor domain. The NadA5 crystal structure was used as a template to generate a homology model of NadA3, which has been used to aid interpretation of epitope mapping studies (30–32). Recently, a panel of 18 human monoclonal antibodies (humAbs) against NadA were isolated from human subjects vaccinated with 4CMenB (32). Interestingly, many of them mediate complement-dependent killing of MenB strains. Most of the humAbs with bacteridical activity target the NadA3 N-terminal head region. Moreover, in an *in vitro* bacterial adhesion/infection assay, humAbs targeting the NadA3 head were able to interfere with binding of NadA-expressing Escherichia coli to Chang epithelial cells (32), suggesting that anti-NadA antibodies might play a role in the inhibiton of meningococcal colonization.

The observations summarized above prompted us to ask “What is the structure of NadA3—the 4CMenB vaccine antigen and prevalent virulence-associated variant—and how do sequence, structural, and biophysical differences relate to functional and immunological differences from NadA5?” Here, we present the X-ray crystallographic structure of the trimeric receptor-binding region of NadA3, which displayed structural features unpredicted by sequence-based methods or homology modeling. We applied sequence- and structure-guided protein engineering and mutagenesis approaches to increase the thermostability of NadA3. We reveal amino acids essential for interactions with the human receptor LOX-1 and diverse protective humAbs elicited by human vaccination, functions which appear to be unique to the group I antigen NadA3.

## RESULTS

### NadA3 residues 24 to 170 mediate stable trimerization.

**FIG 1:**
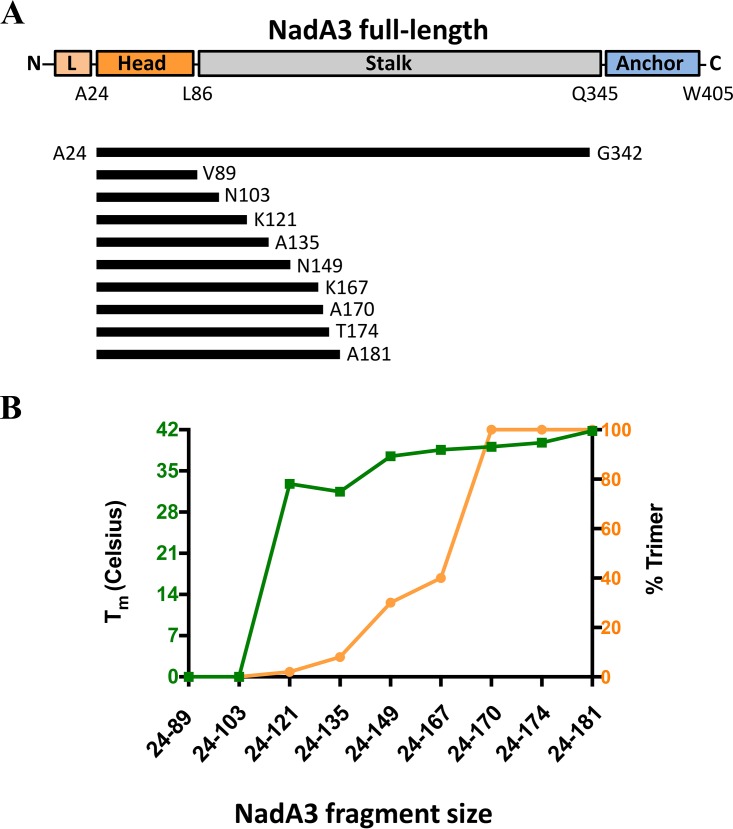
Domain organization and biophysical characterization of NadA3 constructs. (A) Organization of NadA3 constructs; (B) combined data obtained from DSC experiments revealing mean melting temperature (*n* = 2) (left *y* axis, green) and from SEC-MALLS experiments showing the percentage trimer (right *y* axis, orange) for each fragment. L, leader.

The full-length ectodomain of NadA3 is predicted to be highly extended and somewhat flexible, characteristics that may underlie previous crystallization failures (30). To increase NadA3 crystallization likelihood, we focused on N-terminal fragments that are shorter but still harbor functional and immunogenic regions (22, 23, 28, 30, 32). Hence, we sought a trimeric N-terminal fragment with minimal length and maximal stability by screening a panel of seven new NadA3 constructs which all started with residue A24 and extended variously from a maximum of residue A181 to a minimum of residue V89 (Fig. 1A). Tertiary structure in truncated NadA3 proteins was assessed using (i) differential scanning calorimetry (DSC) to measure thermostability and (ii) size exclusion high-performance liquid chromatography (SE-HPLC) coupled to multiangle laser light scattering (SEC-MALLS) to measure molecular size. While all the longer constructs (those containing residues 24 to 149 [NadA3 24–149] or greater) showed similarly high melting point (*T_m_*) transitions, the shorter constructs showed reduced thermotability (lower propensity for a tertiary structure), and indeed the shortest proteins (those containing residues 24 to 89 and 24 to 103) displayed no melting point transition, i.e., lacked a tertiary structure (Fig. 1B).

We then examined the trimeric status of each NadA3 construct, using SEC-MALLS, and observed that the absolute molecular weights (encompassing oligomeric forms) also exhibited a length-dependent pattern. While the three longest fragments (those with residues 24 to 181, 24 to 174, and 24 to 170) were trimeric, the shorter fragments all displayed decreasing proportions of trimer, with fragments 24 to 103 and 24 to 89 being monomeric (Fig. 1B). These DSC and SEC-MALLS data defined the NadA3 24–170 construct as the minimal region exhibiting stable trimerization. The NadA3 24–174 and 24–181 fragments behaved similarly to the NadA3 24–170 fragment, while shorter fragments were only partially trimeric and displayed lower melting temperatures. Additionally, genetic modifications were applied to the NadA3 24–170 construct, attempting further stabilization by fusing the C terminus to the trimerization motif of the yeast GCN4 adaptor or the bacteriophage T4 foldon. While the foldon motif had no discernible effect, the GCN4 motif increased the *T_m_* from 40°C to 45°C (see Fig. S1 in the supplemental material). The GCN4 motif, thus, recapitulates in this truncated protein the stability of the full-length ectodomain of recombinant NadA3, which displayed a *T_m_* of 44°C (30). The NadA proteins studied herein were soluble recombinant forms produced in E. coli; it is conceivable that in meningococci, *in situ*, the additional C-terminal membrane-spanning anchor of NadA confers further stabilization.

10.1128/mBio.01914-18.1 FIG S1 Differential scanning calorimetry (DSC) profiles showing effects of exogenous trimerization motifs on the stability of NadA3 24–170. Genetic fusion of the GCN4 motif to the NadA3 24–170 construct resulted in an increased thermostability (Δ*T_*m*_*, +5°C), while the Foldon motif did not change the *T_*m*_*. Experiments were performed in duplicate (*n *=* *2); for clarity, one representative curve is shown for each sample. Download FIG S1, PDF file, 0.2 MB. Copyright © 2018 Liguori et al. 2018 Liguori et al. This content is distributed under the terms of the Creative Commons Attribution 4.0 International license.

### The crystal structure of NadA3 extends structural understanding.

Numerous crystallization screens were performed using the most stable NadA3 proteins (see Materials and Methods). We determined the crystal structure of NadA3 residues 24 to 170 using the molecular replacement approach and refined the structure to 2.45-Å resolution, with final *R*_work_ and *R*_free_ values of 20% and 22% (Table 1). The asymmetric unit contained three copies of the polypeptide chain. Electron density maps revealed NadA3 residues 28 to 170. The structure presents the typical TAA family protein architecture; it is trimeric and elongated, with an N-terminal globular head. Since the previous NadA5 structure spanned only residues 24 to 137 (30), the NadA3 structure provides new information on an additional 33 residues and, more importantly, on novel folding motifs in NadA.

**TABLE 1:** Data collection and refinement statistics^*a*^

Parameter	NadA3 WT (PDB accession no. 6EUN)	NadA3 A33I-I38L (PDB accession no. 6EUP)
Crystal		
Space group	C 1 2 1	C 1 2 1
		
Cell dimensions		
*a*, *b*, *c* (Å)	69.76, 39.78, 255.49	68.92, 39.75, 255.63
β (°)	95.25	95.13
		
Data collection		
Beamline	ESRF ID23-1	ESRF ID30A-1
Wavelength (Å)	0.977	0.966
Resolution (Å)	34.70–2.45 (2.58–2.45)	63.65-2.65 (2.79–2.65)
Total no. of reflections	58,005 (8,492)	48,983 (6,238)
No. of unique reflections	23,543 (3,487)	18,251 (2,415)
*R*_merge_	0.05 (0.378)	0.08 (0.401)
*R*_meas_	0.062 (0.474)	0.098 (0.494)
*I/*σ(*I*)	9.5 (2.4)	8 (2.2)
*CC*_1/2_	0.998 (0.819)	0.997 (0.850)
Completeness (%)	91.3 (94.2)	89.1 (84.4)
Redundancy	2.5 (2.4)	2.7 (2.6)
Wilson B factor (Å)	51.0	35.3
		
Refinement		
Resolution (Å)	34.84–2.45	63.65–2.65
No. of reflections	23,542	18,248
*R*_work_/*R*_free_	19.9/22.4	19.8/23.6
No. of atoms in:		
Protein	3,250	3,296
Ligand/ion	26	28
Water	52	53
B factors		
Protein	88.27	66.02
Ligand/ion	101.12	70.55
Water	71.61	46.25
RMSD		
Bond lengths (Å)	0.01	0.01
Bond angles (°)	1.14	1.14
Clash scores	2.62	3.29
Ramachandran		
Favored (%)	99.3	99
Allowed (%)	0.7	1

a Values in parentheses are for the highest-resolution shell. $R_{\text{merge}}=\frac{\Sigma_{\text{hkl}}\Sigma_{i=1}^{n}{|I}_{i}\left(\text{hkl}\right)-\overset{¯}{I}\left(\text{hkl}\right)|}{\Sigma_{\text{hkl}}\Sigma_{i=1}^{n}I_{i}\left(\text{hkl}\right)}$ and $R_{\text{meas}}=\frac{\Sigma_{\text{hkl}}\sqrt{\frac{n}{n-1}}\Sigma_{j=1}^{n}\left|I_{\text{hkl,j}}-〈I_{\text{hkl}}〉\right|}{\Sigma_{\text{hkl}}\Sigma_{\text{j}}I_{\text{hkl,j}}}$. *I_i_*(*hkl*) is an individual intensity measurement, and <*I_i_*(*hkl*)> is the average intensity for all the reflections *i*.

**FIG 2:**
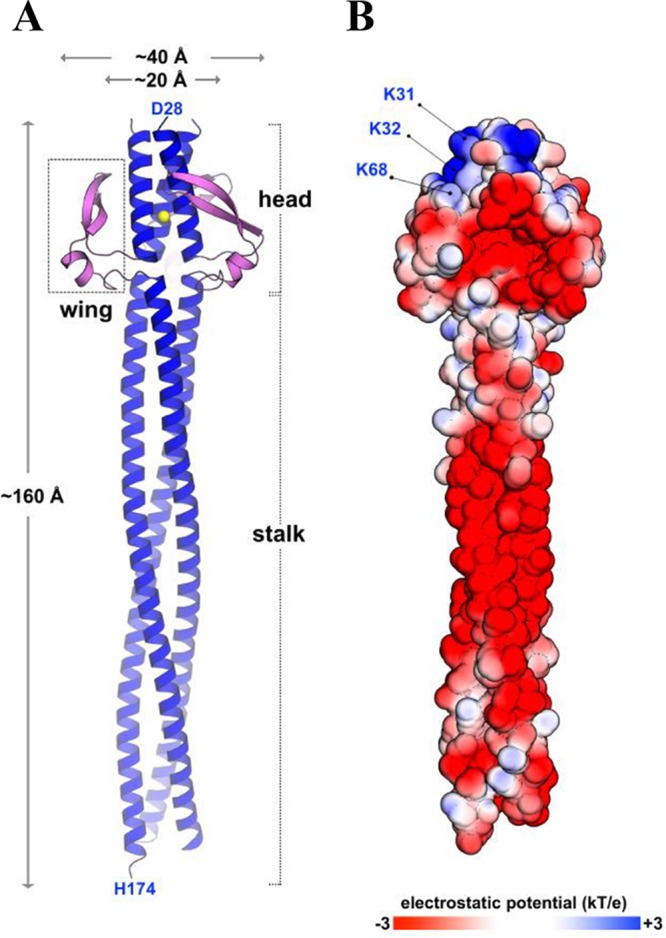
NadA3 displays a head-on-stalk structure and electrostatic charge clusters. (A) Cartoon representation of NadA3 trimer. N- and C-terminal residues are labeled for one chain. A chloride ion (yellow) is buried in the head; wings are pink. Electron density maps lacked four N-terminal residues (_24_ATND_27_) presumably due to local disorder, and revealed four C-terminal residues (_171_ASKH_174_) derived from the hexahistidine tag linker. (B) Electrostatic surface representation of NadA3 (ranging from −3 kT/e [red] to +3 kT/e [blue]), calculated by APBS (adaptive Poisson-Boltzmann solver) methods (57). Artwork was prepared using Pymol (www.pymol.org).

**FIG 3:**
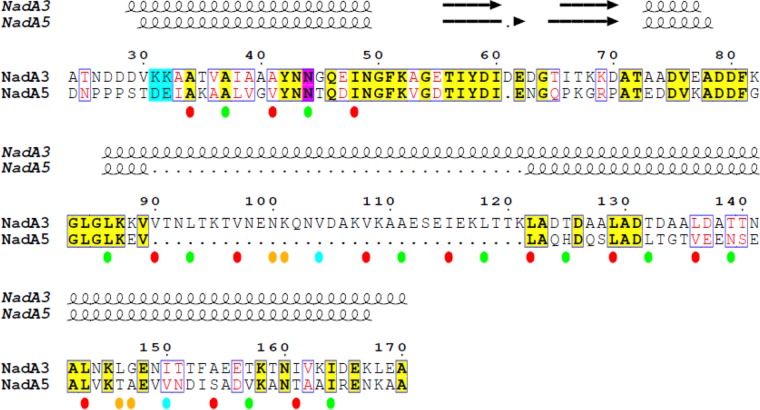
Pairwise structure-based sequence alignment of NadA3 versus NadA5. **(**Above) Secondary-structure elements determined from crystal structures. Numbering corresponds to NadA3. Color code: bold black font on yellow background, sequence identity; red font on white background, sequence similarity; cyan background, contributes to positively charged apex in group I; magenta background, coordinates halide ions. (Below) Red and green ovals highlight heptad repeat positions a and d, respectively. Cyan ovals highlight position h in the undecad repeats. Orange ovals highlight residues forming a *d-e* or *d-a* layer within undecads. The figure was prepared using ESPript 3 (58).

Despite sequence-based predictions that the NadA3 coiled-coil might be interrupted by other secondary structures in the region of residues 100 to 150 (23, 29, 30), the electron density maps of NadA3 24 to 170 display a continuous 160-Å-long coiled-coil stalk region extending from the head all the way to the C terminus (Fig. 2A). Within the head, NadA3 coordinates a chloride ion via the side chain of conserved residue N44, forming an N at the position *d* layer, similar to the observation about NadA5 (30). There is a remarkable distribution of electrostatic potential over the protein surface; while the coiled-coil stalk is negatively charged, the head contains a positively charged apex, largely due to exposure of lysines K31 and K32 (Fig. 2B), which are conserved in NadA1/2 but which display charge inversions to Asp/Glu in the group II antigens NadA4 and NadA5/6 (Fig. 3). Consequently, NadA5 has a less positively charged apex than NadA3.

### Unexpected undecad coiled-coil motifs in NadA3.

**FIG 4:**
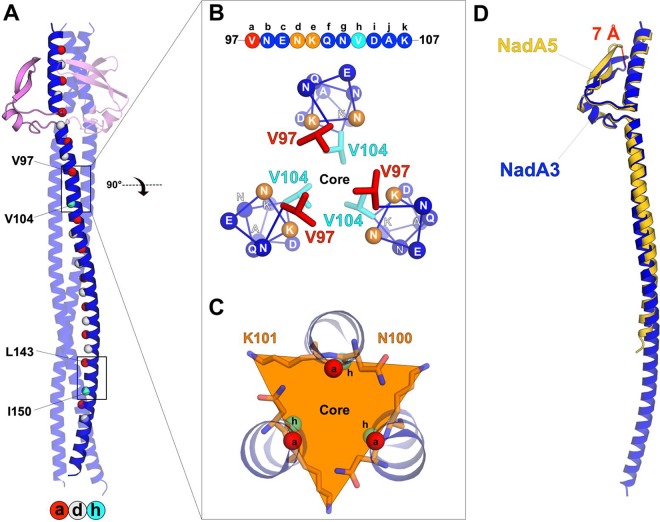
Structure comparisons of NadA3 and NadA5. (A) Locations of 7-residue and 11-residue repeats on NadA3’s coiled coil. Cα atoms in positions *a* and *d* of canonical heptads are red and pale gray spheres, respectively. Black boxes highlight the two undecads. Buried undecad residues *a* and *h* are labeled, and Cα atoms are shown as red and cyan spheres, respectively. (B) Top-view trimeric helical ribbon representations of undecad V97 to K107, showing hydrophobic side chains packed into the core. Positions in the undecad are labeled *a* to *k* (top). Buried *a* and *h* residues are red and cyan sticks, respectively; the remaining Cα atoms are spheres. (C) Cross-sectional triangular layer (orange) in the coiled coil centered at the *d* (N100) and *e* (K101) positions of the undecad. Residues *a* and *h* are spheres, and *d* and *e* residues are sticks. (D) Topology-based superposition of the overall structures of NadA3 (blue) and NadA5 (yellow). The 7-Å difference between the wingtip positions is highlighted. The distance drawn between the wingtips was calculated for corresponding Cα atoms of E62 (NadA3) and E61 (NadA5).

Here, the experimentally determined crystal structure of NadA3 revealed the major differences from NadA5. Two regions in NadA3 unexpectedly contain highly unusual 11-residue (undecad) repeat motifs wherein 11 amino acids form a coiled coil, deviating from the standard heptad repeat pattern (Fig. 4A). Intriguingly, the first undecad repeat spanning V97 to K107 presents an unusual *d-e* layer, previously identified in a few right-handed coiled coils (33–35) and, to our knowledge, never detected before in undecads of left-handed coiled coils. Residues at positions *d* and *e* are less hydrophobic than those in the flanking positions *a* and *h*. Indeed, these two *d-e* residues (N100 [*d*] and K101 [*e*]) are unexpectedly oriented toward the NadA3 surface, resulting in a solvent-exposed triangulated ring of polar residues rather than the typical inward-facing hydrophobic core packing arrangement (Fig. 4B and C). This organization features interhelix van der Waals’ interactions between the *d-e* side chain –CH_2_ groups, likely contributing to coiled-coil stabilization, which is also conferred by the canonical knob-into-hole arrangements at the *a* and *h* positions.

The second undecad repeat, spanning L143 to F153, presents a more canonical hydrophobic *d-a* motif, comprised of residues L146 and G147 (Fig. 3). In this case, the *a* residue is shifted toward the center of the coiled-coil core, while the *d* residue is moved out, resulting in a *d-a* layer, detected previously in other left-handed structures, such as influenza virus hemagglutinin (33, 34). In summary, the NadA3 crystal structure revealed two diverse undecad motifs, unexpected from previous computational analyses due to sequence alignment ambiguities (lack of canonical heptad motifs) that confounded structure predictions (15, 30). Nevertheless, despite sequence variations, the group I and II NadA proteins share a conserved head-on-stalk TAA architecture in their N-terminal portions, without any additional secondary-structure elements.

### NadA heads exhibit minor structural differences.

Overall, the 3D structures of NadA3 and NadA5 are similar. Hence, previous comparisons made only at the sequence level, which left uncertainty about potential interruptions in the coiled-coil of the vaccine antigen NadA3, do not translate into large 3D structural differences. Nevertheless, superpositions of NadA3 and NadA5 reveal different positions of their wingtips by over 7 Å (Fig. 4D). Despite high sequence identity in the head (64%), it is immediately notable that the wing beta-hairpins (residues G54 to T72 in NadA3) have different backbone positions. Moreover, the crystallographic B factors of NadA3 and NadA5 indicate that the head, and especially the wingtips, are the most flexible parts of these structures (Fig. S2).

10.1128/mBio.01914-18.2 FIG S2 Structural similarities and differences of NadA3 and NadA5 head regions. A zoom into the wing regions shows the difference in wingtip positions; optimal superposition was obtained by aligning residues 28 to 48 in both structures with the LSQ algorithm. The wingtips are the most flexible regions of both structures according to B factor analyses. Crystallographic B factors represent temperature-dependent vibrations from average atomic positions. The color bar ranges from low B factor (blue, less flexible) to high B factor (red, more flexible). Download FIG S2, PDF file, 0.03 MB. Copyright © 2018 Liguori et al. 2018 Liguori et al. This content is distributed under the terms of the Creative Commons Attribution 4.0 International license.

### Rationally designed mutations confer increased stability in the NadA3 head.

Despite the high structural similarity, we previously determined that the NadA5 ectodomain (*T_m_*, 58°C) is considerably more stable than the NadA3 ectodomain (*T_m_*, 44°C) (30). To better understand this difference, we sought to identify sites where a NadA5 residue might confer greater stability than the corresponding NadA3 residue. Hence, various mutant NadA3 24–170 proteins with substitutions in the head or stalk regions were prepared and tested for thermostability in DSC experiments. Three single-point mutants exhibited greater thermostability, with *T_m_* values increased by ≥3°C. One mutant had an essentially negligible effect on *T_m_*, and two had a destabilizing effect (*T_m_* decreased by 3°C) (Table 2). Seeking to combine and optimize the stabilizing effects, we made and tested two double mutants. Indeed, strong additive effects were observed, with *T_m_* values increased by 11°C for the A33I A39V double mutant and, remarkably, by 17°C for the A33I I38L double mutant (Fig. S3).

**TABLE 2:** Change in thermostability of NadA mutants compared to wild type *T_m_* of 40°C

NadA protein mutation	Δ*T_*m*_* **compared to that of wild type (°C)^*a*^**
A41V	−3
Y42A	−3
T35K	0
A39V	+3
I38L	+5
A33I	+6
A33I A39V	+11
A33I I38L	+17

a Each DSC experiment was performed in duplicate (*n *=* *2), and mean values of *T_m_* were calculated and compared to that of the wild type.

10.1128/mBio.01914-18.3 FIG S3 Thermostabilization of NadA3 by two point mutations. DSC profiles for native NadA3 24–170 protein (dark red) and the most stabilized double mutant, the A33I I38L construct (orange line), for which the Δ*T_*m*_* measured was 17°C. Experiments were performed in duplicate (*n *=* *2); for clarity, one representative curve is shown for each sample. Download FIG S3, PDF file, 0.2 MB. Copyright © 2018 Liguori et al. 2018 Liguori et al. This content is distributed under the terms of the Creative Commons Attribution 4.0 International license.

**FIG 5:**
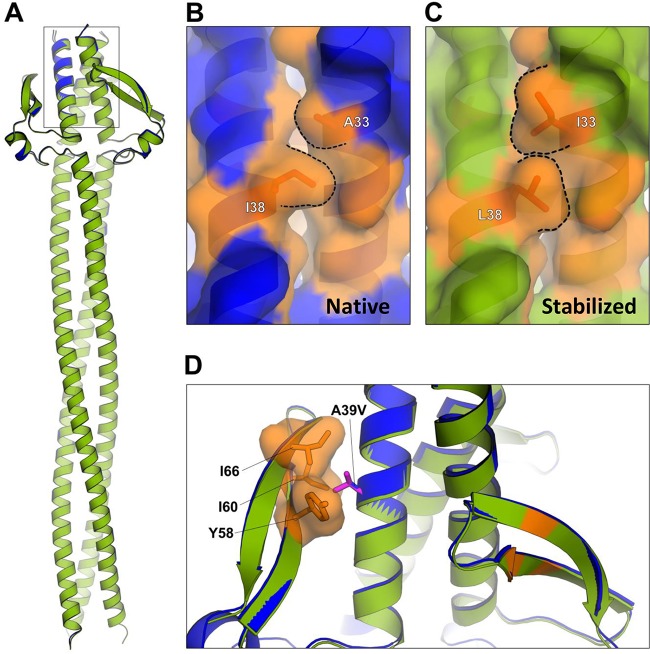
Structure and analysis of stabilized NadA3 mutants. (A) Cartoon representation of superposed native NadA3 24–170 (blue) and the A33I I38L double mutant (green). (B, C) Magnifications of the core region to show the A33I and I38L mutations. Dashes show hydrophobic packing interfaces of residues 33 and 38 in the crystal structures of the native (B) and stabilized double mutant (C) proteins. The interface is more extensive in the double mutant. (D) Magnification of the head proximal to residue A39 (blue stick). Mutation of residue 39 to Val (magenta sticks, as predicted by the mutagenesis tool implemented in Pymol) may increase the extent of favorable hydrophobic packing by filling the cavity lined by adjacent side chains.

Next, we performed structural studies to verify the organization of the most highly stabilized mutant; we crystallized and determined the structure of the NadA3 24–170 A33I I38L double mutant. The structure was refined to a resolution of 2.65 Å, with final *R*_work_ and *R*_free_ values of 20% and 24% (Table 1). Upon superposition, the mutant structure was confirmed to be essentially identical to that of the native protein, even in the relatively flexible wings, exhibiting a root mean square deviation (RMSD) of 0.23 Å for the pairwise superposition of 144 Cα atoms (Fig. 5A). The halide ion, coordinated by Asn44 side chains and >9 Å away from both mutated residues, was present in the same position and with full occupancy in both structures. The halide in the stabilized mutant presented a lower B factor than in the wild type (44.6 Å^2^ versus 69.7 Å^2^), which likely reflects a higher overall stability in the mutant, which has a lower overall B factor than the wild type (66.0 Å^2^ versus 88.3 Å^2^). Moreover, the residues mutated to I33 and L38 present unchanged backbone positions and more-extensive van der Waals’ interactions mediated by their side chains than the original Ala and Ile residues in the native structure (Fig. 5B and C). Therefore, the crystal structure of the double mutant confirmed that improved filling of the cavity with hydrophobic residues had successfully created a stabilized antigen. Although we did not crystallize NadA3 containing the A39V mutation, computational modeling suggests that A39V would also increase thermostability due to its hydrophobic packing, especially against Y58, I60, and I66 (Fig. 5D).

### NadA3 residues A33 and Y42 are crucial for binding to the human receptor LOX-1.

**FIG 6:**
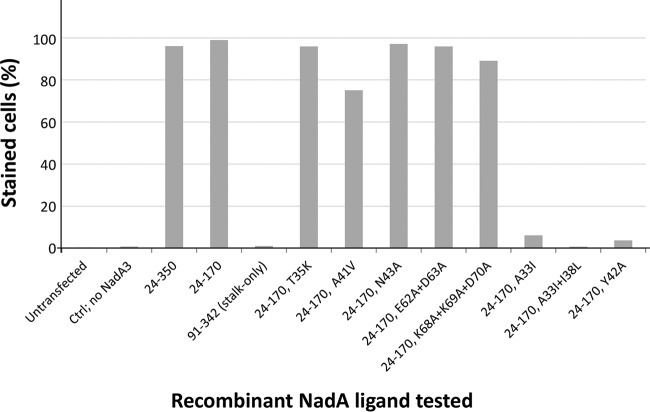
Bar plot summary of flow cytometry data for NadA3 proteins binding to mammalian CHO-K1 cells expressing LOX-1. The bar plot *y* axis shows the percentage of LOX-1-positive transfected CHO-K1 cells that were stained upon incubation with each distinct NadA protein. The NadA3 24–170 proteins carrying point mutations A33I, A33I I38L, or Y42A and the headless-stalk-only protein were heavily impaired for cell binding.

The NadA3 head region was previously implicated in binding the human endothelial cell receptor LOX-1 (28). To identify NadA residues mediating this interaction, we generated several NadA mutants and examined their binding to LOX-1. We used flow cytometry to compare levels of binding of NadA proteins to live mammalian cells transiently expressing full-length LOX-1 (fused genetically to yellow fluorescent protein [YFP]) and to untransfected cells, an assay established previously (28). We confirmed that the trimeric N-terminal fragment of NadA3 (residues 24 to 170, the construct crystallized herein) was sufficient to specifically bind LOX-1-expressing cells in a manner similar to that of the full-length NadA3 ectodomain (residues 24 to 350). In contrast, the NadA3 stalk-only fragment spanning residues T91 to G342, which has a trimeric fold but lacks the head region (31), was unable to bind cells expressing LOX-1 (Fig. 6 and Fig. S4A). We then tested several NadA3 24–170 proteins carrying single or multiple mutations and found that five of the new NadA3 mutants were not impaired for binding to LOX-1, namely, the T35K, A41V, N43A, E62A D63A, and K68A K69A D70A mutants (Fig. 6 and Fig. S4B). However, some mutations abolished binding to LOX-1; these were A33I, A33I I38L, and Y42A (Fig. 6 and Fig. S4C). The single mutation A33I seems to be responsible for most of the loss-of-function in the A33I I38L double mutant, since the double mutant was only slightly more impaired for cell binding than A33I. These findings suggest that NadA3 residues A33 and Y42 play key roles in the interaction with LOX-1 expressed on a live mammalian cell surface.

10.1128/mBio.01914-18.4 FIG S4 Flow cytometry data for NadA3 24–170 proteins binding to mammalian CHO-K1 cells expressing LOX-1. Representative flow cytometry plots are shown. The gating strategy was designed to separate the cells of interest from large aggregates and debris (the initial gate on the forward scatter [FSC] versus side scatter [SSC] plot) and doublets/aggregates (the standard gates on both FSC width and SSC width) (data not shown). Simple gating by quadrants allowed definition of the absolute percentages of cells positive for LOX-1 expression only (LOX-1-transfected cells), cells positive for NadA3 binding only, doubly negative cells (nontransfected cells), and doubly positive cells representing cells able to bind recombinant NadA3 via LOX-1. (A) NadA3 24–170 mediates binding to mammalian cells expressing LOX-1. Untransfected CHO-K1 cells are unable to bind NadA (97.3%). The second panel shows cells positive for LOX-1 expression (55.9%), the third and fourth panels show that only LOX-1-transfected cells are able to bind NadA (53.7% for NadA full-length ectodomain [residues 24 to 350] and 55.3% for NadA 24–170), and the fifth panel shows that LOX-1-transfected cells (55.5%) are unable to bind the NadA stalk (residues 91 to 342). (B) Some mutations in NadA3 24–170 did not affect binding to LOX-1; FACS plots show that LOX-1-transfected cells are able to bind NadA 24–170 mutants (doubly positive cells, 53.6% for the T35K mutant, 42% for the A41V mutant, 54.3% for the N43A and E62A D63A mutants, and 49.8% for the K68A K69A D70A mutant). (C) Some mutations in NadA3 24–170 abolished binding to LOX-1; plots show that LOX-1-transfected cells are unable to bind specific NadA3 24–170 mutants (no doubly positive cells were observed), namely, the A33I, A33I I38L, and Y42A mutants. Download FIG S4, PDF file, 0.4 MB. Copyright © 2018 Liguori et al. 2018 Liguori et al. This content is distributed under the terms of the Creative Commons Attribution 4.0 International license.

**FIG 7:**
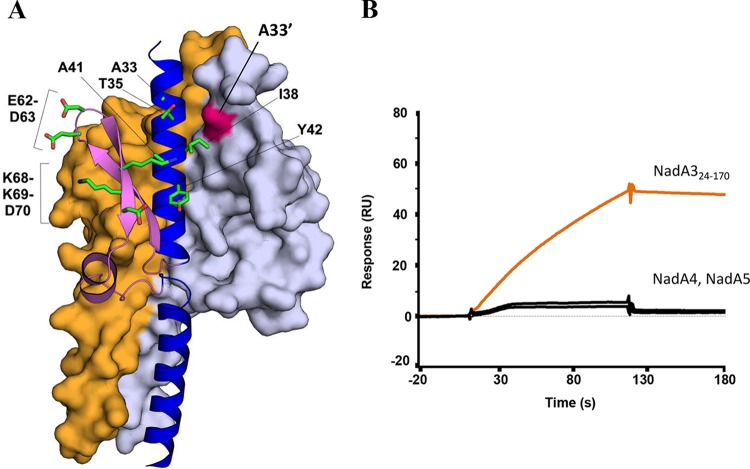
Sites of mutagenesis and NadA–LOX-1 interaction specificity. (A) Cartoon and surface representations of the trimeric-head region of NadA3, showing on one chain the side chain sticks for the subset of residues selected for mutation and on an adjacent chain the surface corresponding to residue A33′ (in pink). (B) SPR single-injection experiments showing binding of immobilized LOX-1 to injected NadA3 (orange line), but not to either NadA4 24–219 or NadA5 24–220 (black lines). Each protein (analyte) was injected in duplicate (*n = 2*); for clarity, one representative sensorgram is shown for each protein.

Further, we used surface plasmon resonance (SPR) to quantify the NadA–LOX-1 ectodomain binding affinity. The SPR data revealed a high-affinity interaction between LOX-1 and NadA3 24–170, with an equilibrium dissociation constant (*K_D_*) of ∼2 nM (Fig. S5), in agreement with the data obtained previously using a biolayer interferometry assay (28). In order to quantify the binding to LOX-1 demonstrated by a weak binder in the fluorescence-activated cell sorter (FACS) experiments (Fig. 6), we performed an SPR titration using the NadA3 Y42A mutant, which revealed a *K_D_* of ∼4 μM, approximately 1,000-fold larger than that of wild-type NadA3 (Fig. S5). In summary, the flow cytometry and SPR data were effective in screening a panel of mutants and highlighted the importance of NadA3 residues A33, I38, and Y42 in binding to LOX-1. Moreover, a structure-based inspection of the locations of the NadA3 loss-of-function mutations suggests that LOX-1 binds in a crevice between monomers of the trimer, likely involving residues I38 and Y42 of one chain and A33′ of the adjacent chain (Fig. 7A).

10.1128/mBio.01914-18.5 FIG S5 SPR titrations were used to determine the equilibrium dissociation constants (*K_*D*_*), by injecting multiple increasing concentrations of NadA3 proteins over a sensorchip on which LOX-1 was captured. The Y42A mutant (right; *K_*D*_*, 3.9 ± 0.2 mM) showed approximately 1,000-fold-weaker binding than the native protein (left; K*_*D*_*, 1.8 ± 1 nM). The injection at each concentration was performed in duplicate (*n *=* *2), and both experimental curves are shown as colored lines; the black line shows the calculated fit. Download FIG S5, PDF file, 0.2 MB. Copyright © 2018 Liguori et al. 2018 Liguori et al. This content is distributed under the terms of the Creative Commons Attribution 4.0 International license.

Interestingly, while both A33 and Y42 seem particularly important for binding to LOX-1, only Y42 is conserved (99%) in all group I and II NadA proteins, whereas A33 is conserved only in group I (Fig. 3). To explore possible functional differences, we used SPR to compare the specificities of LOX-1 in binding to NadA variants from groups I and II. We found that the group II variants NadA4 and NadA5 showed only very weak interactions with LOX-1 (Fig. 7B), suggesting that the interaction is specific for NadA3 (group I). While the presence of the bulky Ile side chain at position 33 in NadA4 and NadA5 may confer greater thermostability, it may also cause the functional closure of this intermonomer pocket, resulting in the observed loss of binding to LOX-1.

### NadA3 elicits human antibodies that bind protective epitopes in the head region.

**FIG 8:**
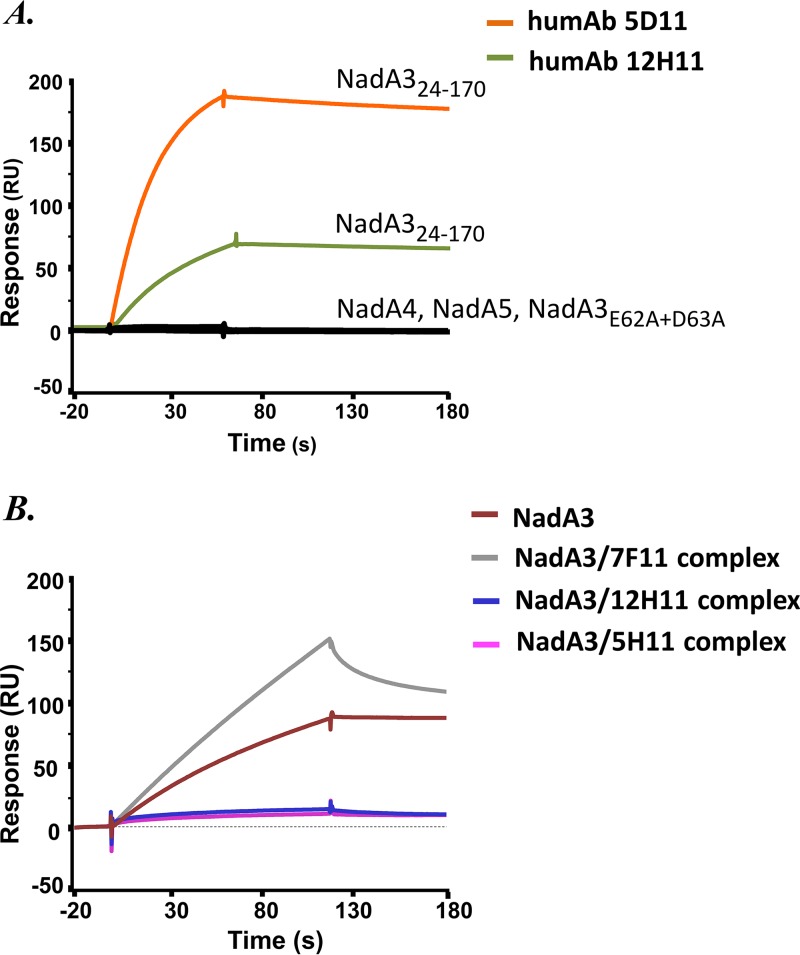
Affinity and selectivity of the NadA–LOX-1–humAb interactions. (A) SPR single-injection experiments, showing that both humAbs 5D11 and 12H11 bind to NadA3 but not to NadA4 24–219, NadA5 24–220, or the NadA3 E62A D63A double mutant. (B) SPR single-injection experiments. Injected NadA3 binds to immobilized LOX-1 but not if NadA is preincubated with humAb 12H11 or 5D11. Preincubation with humAb 7F11 (an anti-stalk humAb) does not abolish binding to LOX-1. Each protein (analyte) was injected in duplicate (*n* = 2); for clarity, one representative sensorgram is shown for each protein.

Eighteen anti-NadA human monoclonal antibodies (humAbs) raised by immunization with the 4CMenB vaccine were recently characterized (32). Eight of the 18 humAbs were found to bind epitopes in the NadA3 head region spanning residues 52 to 75. Two of these humAbs, 5D11 and 12H11, were of particular interest, since they were found to bind with very high affinity to NadA3 (*K_D_* < 50 pM), with their epitopes mapped to include a short linear stretch in the wingtips (61-DEDGTIT-67) as identified via a synthetic-peptide-based method (while such short epitopes were not identified for the other antihead humAbs) (32). Despite targeting an apparently small epitope, these two humAbs were highly functional, i.e., exhibited strong bactericidal activity (32), suggesting that they may target a minimal protective epitope in NadA3, which might be of interest to aid an understanding of immunogenicity and potentiate antigen design. Indeed, when tested in the standard serum bactericidal assay, humAbs targeting the head generally exhibited higher bactericidal titers than those targeting the stalk. Moreover, only humAbs targeting the head were effective at inhibiting NadA-mediated adhesion to Chang epithelial cells (32). To delineate these protective and functional immune responses, we sought to identify key target epitope residues for humAbs 5D11 and 12H11. In SPR analyses using the NadA3 mutants described above (Fig. 7A), only the E62A D63A double mutant demonstrated reduced binding to the two humAbs tested, namely, 5D11 and 12H11 (Fig. 8A). These two humAbs share 72% sequence identity in their heavy-chain variable regions and 82% identity in their light-chain variable regions. Homology models of these humAbs show that their paratopes contain several large positively charged surface patches (Fig. S6), which appear well suited to mediate their interactions with a negatively charged epitope, likely involving NadA3 residues E62 and D63. Interestingly, neither of these humAbs were able to bind NadA4 or NadA5. While NadA3 here contains three charged residues, 61-DED-63, in contrast, NadA5 contains only one glutamate (Fig. 3), which may underlie its lack of binding to these two humAbs, thus providing insights into variant-specific humAb responses.

10.1128/mBio.01914-18.6 FIG S6 Homology models of the anti-NadA humAbs tested herein, 12H11 (A) and 5D11 (B), both of which expose several positively charged residues in their paratopes. Download FIG S6, PDF file, 0.1 MB. Copyright © 2018 Liguori et al. 2018 Liguori et al. This content is distributed under the terms of the Creative Commons Attribution 4.0 International license.

### Vaccine-elicited antibodies against NadA3 inhibit binding to human receptor LOX-1.

The Y42A mutation, which reduced the binding of NadA3 to LOX-1, had essentially no impact on the binding of NadA3 to the humAbs. Nevertheless, since Y42 and E62/D63 are separated by a relatively short distance (Fig. 7A), we sought to determine whether the binding of NadA3 to LOX-1 and the humAbs was mutually exclusive. Indeed, LOX-1 immobilized on the SPR sensor chip surface bound efficiently to injected NadA3, but preincubation of NadA3 with humAb 5D11 or 12H11 was competitive and eliminated the interaction with LOX-1 (Fig. 8B). As a control, the preincubation of NadA3 with humAb 7F11, which targets the stalk region of NadA (32), did not reduce the binding of NadA to LOX-1 (on the contrary, an increased SPR response was observed, due to binding of the larger NadA 7F11 complex to immobilized LOX-1). Under these conditions, NadA3 bound to all three humAbs with subnanomolar affinity (Fig. S7). In summary, humAbs binding to NadA3 can compete the binding of NadA to LOX-1, suggesting that vaccine-elicited humAbs may interfere with the meningococcal/human host-pathogen interactions at the endothelium.

10.1128/mBio.01914-18.7 FIG S7 Sensorgrams from SPR single-cycle kinetics experiments with injection of NadA3 over captured human mAbs 5D11 (A), 12H11 (B), and 7D11 (C). Each titration was performed in duplicate (*n *=* *2), and both experimental curves are shown as colored lines; the black line shows the calculated fit. Download FIG S7, PDF file, 0.2 MB. Copyright © 2018 Liguori et al. 2018 Liguori et al. This content is distributed under the terms of the Creative Commons Attribution 4.0 International license.

## DISCUSSION

N. meningitidis causes invasive meningococcal disease and may result in devastating sequelae or death (1, 2). In 2013, approval was granted for the first broadly protective vaccine against serogroup B N. meningitidis, a multicomponent formulation termed 4CMenB (or Bexsero). The 4CMenB vaccine contains an outer membrane vesicle component plus three main recombinant protein antigens, including the neisserial adhesin A variant 3 (NadA3) (7, 11). NadA is a surface-exposed meningococcal virulence factor and a powerful antigen able to induce bactericidal antibody responses in humans in all age groups. Six variants have been identified and classified in two groups, I and II. NadA3, present in the 4CMenB vaccine, belongs to group I. The crystal structure of NadA5, belonging to group II, was reported previously (30). However, NadA5 shares only 46% to 50% sequence identity with NadA3, and they are not immunologically cross-reactive (16). We performed a variety of investigations to explore the structural, functional, and antigenic differences between NadA5 and the 4CMenB antigen NadA3. We also characterized key epitopes recognized by functional vaccine-elicited humAbs and their involvement in competing with the binding of NadA3 to the human receptor LOX-1.

First, we performed biophysical studies of the thermostability and trimerization of several NadA3 N-terminal fragments. Although these two properties of the proteins were not directly correlated, they presented similar trends indicating that shorter N-terminal fragments were less stable and allowed identification of the construct NadA3 24–170 as the shortest fragment consistently stable as a trimer and with essentially the maximal thermostability. This construct ultimately yielded crystals sufficient to determine the structure of NadA3.

Although the new NadA3 structure was overall similar to NadA5, it presented notable differences in the wingtip positions and two unexpected 11-residue (undecad) coiled-coil repeats. Generally, undecads and other heptad discontinuities can cause local instability by introducing flexibility (36, 37), but the relative impact of this on protein stability remains unclear when considered alongside other coil properties, such as helical propensity, core hydrophobicity, core packing, solvent shielding, and other factors. Indeed, stability can be maintained when the insertion of the undecad is combined with adjustments of the coiled coil that permit the canonical knob-into-hole configuration, as observed for *de* and *da* layers. In both such cases, as reported here for NadA3, and also previously for Omp α from T. maritima (38), residues in positions *d*, *e* and *d*, *a* form rings of interactions around a central core, which may retain protein stability. Overall, the structural roles of undecads in coiled-coil proteins are still poorly understood and warrant further investigation on a case-by-case basis. In NadA3, disruption of the undecad repeats likely underlies the poor stability of the shorter NadA3 constructs initially tested herein. In order to explore this, future studies might attempt the generation of domain-swapped forms of NadA3 and NadA5 in the regions containing the undecad repeats. This experimental structure determination revealed features that even well-established coiled-coil prediction servers had not been able to detect, thus providing new empirical structural data that potentially strengthens the training of existing coiled-coil structure prediction algorithms. The structure of NadA3 24–170 presents a small globular head region (residues 24 to 85) and a long trimeric coiled-coil motif made of canonical heptad repeats, plus the two novel undecad repeats. Sequence analyses of the noncrystallized ectodomain region from residues 171 to 345 suggest the presence of several additional heptad repeats and perhaps another undecad repeat. It remains very challenging to obtain a reliable sequence-based prediction of the structure of the central region spanning residues 250 to 280 of NadA3.

The vaccine antigen NadA3 was much more challenging to crystallize than NadA5. Although the NadA3 antigen can be produced and purified in a consistent manner and is physico-chemically very stable, it shows a lower thermostability (*T_m_*, 44°C) than NadA5 (*T_m_*, 58°C), making the formation of crystals much simpler in the case of NadA5. Through a cavity-filling mutagenesis approach on NadA3, we identified the mutations A33I, I38L, and A39V, all of which increased thermostability, with the A33I I38L double mutation conferring a remarkable 17°C *T_m_* increase. It would be interesting to determine whether the corresponding reverse mutations (i.e., I33A and L38I) could confer a destabilizing effect on NadA5; molecular modeling suggests that this would be the case (see Fig. S8 in the supplemental material). The crystal structure of this double mutant confirmed that an improved packing of the hydrophobic core around residues 33 and 38 had been achieved and that its overall conformation was essentially unperturbed compared to that of the wild type. We performed these protein-stabilizing engineering studies, informed by the crystal structures of NadA3 and NadA5, using the N-terminal fragment of NadA3 spanning residues 24 to 170, which is emerging as the most interesting region of NadA, both from a functional and an antigenicity viewpoint (see below). Indeed, these data may aid the future design of improved immuno-focused vaccine antigens, since in some cases protein stability has been positively correlated with the quality of immune response (39).

10.1128/mBio.01914-18.8 FIG S8 (A and B) Semitransparent surface plots of the experimental NadA5 crystal structure (A) and the *in silico* model harboring mutations I33A and L38I (B); (C and D) same molecular views but with opaque surface rendering. The images serve to illustrate that in wild-type NadA5 the residues I33 and L38 make greater Van der Waals’ interactions (indicated by black dashed lines) than would be predicted for the NadA5 double mutant, suggesting that the latter would be destabilized compared to the wild type. Download FIG S8, PDF file, 0.1 MB. Copyright © 2018 Liguori et al. 2018 Liguori et al. This content is distributed under the terms of the Creative Commons Attribution 4.0 International license.

We also used structure-guided site-directed mutagenesis, coupled with surface plasmon resonance (SPR) and flow cytometry analysis, to identify residues important for interactions of NadA3 with humAbs and with the human endothelial receptor LOX-1, one of several human proteins reported to interact with NadA. For example, in addition to NadA and the external Hsp90, which have been demonstrated to interact (24–26), LOX-1 and β1 integrin are two human putative receptors identified to date (28), and other host molecules recognizing the adhesin are likely to exist. Indeed, NadA binds to Chang epithelial cells, which do not express LOX-1, in a β1 integrin-independent manner and also bind to purified monocytes and THP-1 human monocytic cells independently of LOX-1 and β1 integrin (M. Merola and B. Benucci, unpublished data). In all instances reported to date, the membrane-distal N-terminal head region of NadA and the minimum stalk region required to maintain the trimeric organization have been identified as the crucial regions for receptor binding specificity (22; also this study). The ability of NadA to recognize multiple targets is one of the features shared with the other members of the trimeric autotransporter family (21, 40). The NadA3 residues A33 and Y42 were crucial for the interaction with LOX-1 and are colocated in an intermonomer crevice of the trimer. An experimentally determined structure of the NadA–LOX-1 complex is required to fully clarify the mode of binding. We also demonstrated that the interaction with LOX-1 was specific for NadA3 but that the group II variants NadA4 and NadA5 did not show appreciable binding. The pattern of binding specificity and selectivity was confirmed in a second, more physiologically relevant context, i.e., binding of NadA to mammalian cells expressing LOX-1. It is interesting that the group II variants tend to be associated with carrier strains rather than invasive disease-causing strains (16). It has been reported previously that LOX-1 is involved in mediating adhesion of Gram-negative and -positive bacteria (41); further investigations are required to determine whether the interaction of NadA with LOX-1 may be a mechanism that confers increased virulence to meningococcal strains expressing group I variants.

Finally, we studied the interactions of NadA with a subset of vaccine-elicited anti-NadA humAbs. We found a double mutant (NadA3 E62A D63A) that exhibited reduced binding to the humAbs 5D11 and 12H11, suggesting that the wingtips at the top of the NadA3 head are essential for antibody binding. As seen for the interaction with LOX-1, both humAbs were specific for NadA3, and very little binding was observed for NadA4 or NadA5. These results suggest that this exposed head region contains at least some of the key residues that underlie the lack of cross-reactive immunogenicity and likely the receptor specificity of the NadA group I and II proteins. Interestingly, we also observed that the NadA3 region involved in binding to LOX-1 is close to the epitopes recognized by the two humAbs studied herein. Both of these humAbs are able to efficiently mediate complement-dependent killing of meningococci expressing NadA3 (32). Moreover, we have shown *in vitro* that humAbs 5D11 and 12H11 directly compete with the NadA–LOX-1 interaction, and we speculate that this mutually exclusive binding may inhibit *in situ* the NadA-mediated adhesion of meningococci to human cells.

Our earlier preclinical epitope-mapping studies on NadA have shown that a bactericidal mouse MAb (33E8) recognizes a group I-specific epitope in the NadA head domain (30). Intriguingly, the MAb 33E8 conformational epitope involves peptides V36 to G50 and I60 to A74, encompassing the residues Y42 and E62/D63, also identified here as key residues for the interactions with LOX-1 and humAbs. Our studies show that this functionally important N-terminal region of NadA is immunogenic in both mice and humans, and vaccine-elicited MAbs targeting this important epitope are protective against MenB. Future antigen design efforts might seek specifically to direct the immune response toward this epitope, akin to the immunofocusing approaches explored for a respiratory syncytial virus vaccine (42).

In summary, we determined the structure of NadA3, a key antigen of the multicomponent 4CMenB vaccine antigen now in widespread use, in order to better understand its antigenicity and function. Crystal structures of the native NadA3 antigen and of a thermostabilized double mutant provided numerous new insights into the fold of this protein. In addition, biochemical and cell-based assays revealed amino acids crucial for the functional interactions of NadA3 with the human receptor LOX-1 and with vaccine-elicited humAbs. Collectively, these insights provide an improved characterization of a 4CMenB vaccine component and of its biological mechanism of action. We expect that such structural and functional data will provide a useful platform both for the continued interpretation of the human immune response to 4CMenB vaccination and for further investigations of host-pathogen interactions influencing meningococcal virulence.

### Clinical samples.

The human antibodies studied were obtained via a phase I clinical trial conducted in Krakow, Poland, approved by the Bioethics Committee of the District Medical Doctors’ Chamber in Krakow and conducted in accordance with the Declaration of Helsinki. Samples were used after written informed consent was obtained from participants before the study-specific procedures. We thank the clinical study participants.

## MATERIALS AND METHODS

### Cloning and site-specific mutagenesis.

Construct design was guided by the NadA5 crystal structure, and it was observed that the NadA3 construct A24-V89 is unfolded, whereas NadA3 A24-A170 is folded (30, 31). Gene fragments of *nadA* were PCR amplified from the N. meningitidis serogroup B strains 2996 (NadA3), NGE28 (NadA4), and M01-240320 (NadA5) and were inserted into the pET-21b(+) expression vector (Novagen), as described previously (30). *nadA* expression constructs were cloned without signal peptides and with a hexahistidine (6×-His) tag at the C terminus. Sequence numbering refers to the full-length NadA3, NadA4, and NadA5 proteins with UniProt codes Q8KH85, Q5J6N1, and A0ELI2, respectively. Subfragments and point mutants were prepared using polymerase incomplete primer extension (PIPE) cloning methods (43). The additions of GCN4 or fibritin sequences to the NadA3 24–170 construct were performed via gene synthesis (Geneart). The GCN4 motif comprised 28 amino acids (four successive heptads), was designed according to the GCN4/SadA structure (44), and was added in register to the A24-to-A170 construct followed by a 6×-His tag. The foldon motif comprised 27 amino acids (GYI…TFL) from the C terminus of the collagen-foldon structure (45) and was separated from NadA by a short GlyGlySer linker and followed by a 6×-His tag.

### Protein production and purification. (i) NadA proteins.

Escherichia coli strain BL21(DE3) cells (Novagen) were used for protein expression. Cells were grown using BioSilta medium (Enpresso B animal-free growth systems), at 30°C for 18 h, upon which NadA production was induced by addition of 0.1 mM IPTG (isopropyl β-d-thiogalactopyranoside). After an additional 24-h culture, cells were harvested by centrifugation and were suspended in 50 mM NaH_2_PO_4_, 300 mM NaCl, pH 8.0, followed by lysis via sonication (Sonica Q700). The clarified E. coli extract supernatant containing soluble NadA protein was loaded on a 1-ml HisTrap column (GE Healthcare) for purification by Ni^2+^ affinity chromatography. Proteins were eluted in buffer containing 50 mM NaH_2_PO_4_, 250 mM NaCl, 250 mM imidazole, pH 8.0. Eluted fractions containing NadA (identified by SDS-PAGE) were pooled and further purified by size exclusion chromatography (SEC) using HiLoad (16/60) columns (Superdex 75 or Superdex 200 [for the larger constructs]) (GE Healthcare) equilibrated in 20 mM Tris-HCl, 150 mM NaCl, pH 8.0. Overall, this process typically yielded 1 mg purified protein per g wet biomass. Purified proteins were used immediately for crystallization trials or were frozen for storage at −20°C.

### (ii) Antibodies.

The recombinant human monoclonal antibodies (humAbs) were cloned and produced by transient transfection of mammalian Expi293 cells (ThermoFisher Scientific) and were purified from the supernatant via protein G affinity chromatography (GE Healthcare) as described previously (32).

### DSC.

The thermostability of NadA proteins was assessed by differential scanning calorimetry (DSC) using a MicroCal VP-capillary DSC instrument (GE Healthcare). NadA proteins were prepared at a concentration of 0.5 mg/ml (∼10 μM trimer) in phosphate-buffered saline (PBS). The temperature scan ranged from 10°C to 110°C, with a thermal ramping rate of 200°C per h. Data were analyzed by subtraction of the reference buffer data, using the Origin 7 software. All experiments were performed in duplicate, and mean values of the melting temperature (*T_m_*) were determined.

### SE-HPLC coupled to MALLS.

Size exclusion high-performance liquid chromatography (SE-HPLC) was performed at room temperature (18 to 26°C) on an analytical size exclusion TSK Super SW3000 column (Tosoh) equilibrated in buffer containing 10 mM NaH_2_PO_4_, 400 mM (NH_4_)_2_SO_4_ pH 6.0, loading 40 μg of NadA sample. Eluted fractions containing NadA were immediately analyzed by multiangle laser light scattering (MALLS) analyses performed using a Dawn TREOS MALLS detector (Wyatt) and an incident laser wavelength of 658 nm. The intensity of the scattered light was measured at 3 angles simultaneously. Data analyses were performed using Astra V software (Wyatt). Normalization of the MALLS detectors was performed within each analytical session by use of a bovine serum albumin (BSA) sample.

### Protein crystallization.

Purified NadA proteins were concentrated to between 10 and 42 mg/ml, depending on the construct, and were subjected to crystallization trials using a Crystal Gryphon liquid handling robot (Art Robbins Instruments). Crystallization screening experiments were prepared by mixing equal volumes (200 nl) of a NadA sample with crystallization reservoir solution by using five different commercially available screens (JCSG-Plus, Morpheus, and Structure, from Molecular Dimensions, Ltd., and PEGIon and SaltRX from Hampton Research). Crystal growth was attempted at 4°C and 20°C in a sitting-drop vapor diffusion format using 96-well low-profile Intelliplates (Art Robbins Instruments).

Only the NadA3 A24–A170 protein yielded single and sizable crystals, obtained at 20°C, using reservoir condition B6 of the JCSG-Plus HT-96 screen (Molecular Dimensions, Ltd.), which contains 0.1 M sodium phosphate citrate, pH 4.2, 40% (vol/vol) ethanol, and 5% (wt/vol) polyethylene glycol 1K (PEG 1K). Over 100 such crystals were tested but yielded only poor X-ray diffraction. Optimization using alcohols with lower volatility was required to reproducibly obtain diffraction-quality crystals, using a reservoir containing 0.1 M sodium phosphate citrate, pH 3.9, 5% (wt/vol) PEG 1K, and 2-methyl-2,4-pentanediol (MPD) in a concentration range of 33.6% to 45.9% (vol/vol) and NadA at a concentration of 4 mg/ml. NadA crystals were mounted in cryo-loops without additional cryo-protectant and were stored in liquid nitrogen prior to data collection.

### Structure determination.

X-ray diffraction data were collected at cryogenic temperatures (100 K) on beamlines ID23-1 and ID30A-1 at the European Synchrotron Radiation Facility (ESRF), Grenoble, France, using a Dectris Pilatus 6M-F (NadA3 wild type) and a Dectris Pilatus3 2 M (NadA3 A33I-I38L mutant) detector. Diffraction data were indexed and integrated with Mosflm 7.2.1 (46) and scaled with the software Scala (47) from the CCP4 suite (48).

The crystals of wild-type NadA3 belonged to the monoclinic *C*2 space group, with the following cell parameters: *a *=* *69.8 Å, *b *=* *39.8 Å, *c *=* *255.5 Å, and β angle = 95.2°. A Matthews coefficient of 3.5 Å^3^/Da, compatible with a solvent content of 65%, suggested the presence of one trimer in the asymmetric unit. The structure was solved by molecular replacement in Molrep (49) using the structure of NadA5 (PDB code 4cjd) as the search model. Manual model building and refinement were performed with Coot (50) and BUSTER (51), respectively. The structure of the mutant NadA3 A33I–I38L was solved in space group *C*2 by molecular replacement by using one trimer of the NadA3 24–170 refined coordinates as the search model.

### Structure analysis.

The final models were inspected and validated using Molprobity (52). Structure solution and refinement statistics are reported in Table 1. All figures were generated using the molecular graphic software Pymol (PyMOL Molecular Graphics System, version 1.8; Schrödinger, LLC; http://www.pymol.org). The topology-based alignment and the least-squares superpositions were performed with SSM and LSQ algorithms, both implemented in Coot (53).

### Surface plasmon resonance.

Surface plasmon resonance (SPR) was used to characterize interactions of NadA proteins either with the LOX-1 receptor ectodomain (R&D Systems) or with a panel of recombinant humAbs (32). Experiments were performed at 25°C in running buffer containing 10 mM HEPES, pH 7.4, 150 mM NaCl, 2 mM CaCl_2_, 0.05% (vol/vol) Surfactant P20 supplemented with 1% BSA to minimize nonspecific interactions.

#### NadA–LOX-1 binding studies.

In the direct binding assay, LOX-1 was immobilized on a carboxymethylated dextran sensor chip (CM-5; GE Healthcare) to a level of approximately 2,000 response units (RU). An unmodified surface was used as the reference channel. For single-injection experiments, the NadA protein samples were injected at a 100 nM concentration over the surface at a flow rate of 30 µl/min for 120 s, followed by a dissociation time of 120 s. To remove the remaining nondissociated samples, the surface was regenerated with a 5-s injection of 10 mM glycine-HCl buffer, pH 1.7, at 30 µl/min (GE Healthcare). For determination of the equilibrium dissociation constant (*K_D_*) and kinetic rate constants for LOX-1 binding to wild-type NadA3 24–170 and the NadA3 Y42A mutant protein, a multiinjection titration series of five consecutive injections (at 40 μl/min for 120 s) of purified NadA protein was performed with increasing concentrations (7.8 to 125 nM for the wild type and 0.25 to 4 µM for the Y42A mutant). Surface regeneration was performed as described above.

#### NadA–LOX-1–antibody competition studies.

The same CM-5 sensor chip as above was used with the ligand LOX-1 covalently immobilized. Prior to injection, the NadA analyte at a 50 nM concentration was incubated with humAb at a 75 nM concentration for 1 h at 4°C. The chip surface was regenerated with a 120-s injection of regeneration buffer (10 mM glycine-HCl, pH 1.7), at a flow rate of 10 μl/min.

#### NadA-antibody binding studies.

For the single-cycle kinetics (SCK) experiments with humAbs, a commercially available human antibody capture kit (GE Healthcare) was used to immobilize anti-human IgG antibodies by amine coupling to a carboxymethylated dextran sensor chip. An IgG density level of ∼7,000 RU was achieved. The immobilized anti-human IgG was then used to capture ∼1,000 RU of the anti-NadA humAbs. An anti-human IgG antibody-coated surface without captured MAbs was used as the reference channel. NadA samples were injected at multiple increasing concentrations (0.625, 1.25, 2.5, 5, and 10 nM) with a contact time of 80 s at a flow rate of 30 µl/min. The final dissociation phase was monitored for 600 s, and the chip surface was regenerated between each consecutive cycle as described above.

All SPR experiments were performed in duplicate using a Biacore T200 instrument (GE Healthcare). A blank injection of buffer only was subtracted from each binding curve, and reference sensorgrams were subtracted from experimental sensorgrams to yield curves representing specific binding. SPR data were analyzed using the Biacore T200 evaluation software (GE Healthcare). For all the SCK experiments, each sensogram was fitted with the 1:1 Langmuir binding model, including a term to account for potential mass transfer, to obtain the individual *k*_on_ (association rate) and *k*_off_ (dissociation rate) kinetic constants; the individual values were then combined to derive the single averaged *K_D_* values reported (54).

### Flow cytometry assays.

The binding assay was performed as described previously (28). Briefly, CHO-K1 cells (ATCC CCL-61) were transfected with a pEYFP-N1 vector expressing human LOX-1 and were allowed to recover for 48 h. Cells were nonenzymatically detached using cell dissociation solution (CDS; Sigma), harvested, washed in PBS, and resuspended in F12K medium (Gibco) supplemented with 1% inactivated fetal bovine serum (iFBS). Approximately 3 × 10^5^ cells were mixed with native or mutant NadA3 forms (all of the same length, spanning NadA3 A24–A170) (200 µg/ml) or blocking solution (PBS plus 1% iFBS) alone for 30 min at 37°C. Cells were then incubated with mouse polyclonal anti-NadA3 (1:1,000 dilution) for 1 h at 4°C and with allophycocyanin (APC)-conjugated goat F(ab)2 antibody to mouse Ig (diluted 1:100; Thermo Scientific) for 30 min at 4°C. Cells were analyzed with a Canto II analyzer (Becton, Dickinson, Pharmingen, San Diego, CA). Flow cytometry data were analyzed using FlowJo software (Treestar Inc.).

### Homology modeling of antibody F(ab) fragments.

Sequences of the F(ab) fragment variable regions from humAbs 12H11 and 5D11 were used for homology detection in the molecular operating environment (MOE) (55), and the top template structures with the highest scores were selected and used for subsequent 3D homology model building using the antibody modeller function in MOE. The top hits found were PDB accession no. 5JOF (VRC03 gHVgLV antigen-binding fragment) for F(ab) 12H11 and PDB accession no. 4HWB (human neutralizing antibody fragment in complex with the ectodomain 3 of the IL-13 receptor alpha 1) for F(ab) 5D11. The CDRs (complementarity determining regions) were then automatically annotated from the sequence of the newly generate models using Kabat’s numbering scheme.

### Accession number(s).

The final coordinates and structure factors of wild-type NadA3 and the A33I I38L NadA3 double mutant have been deposited in the Protein Data Bank with accession codes 6EUN and 6EUP, respectively.
