# Supplementary material for: NadA3 Structures Reveal Undecad Coiled Coils and LOX1 Binding Regions Competed by Meningococcus B Vaccine-Elicited Human Antibodies
Source: mBio. 2018 Oct 16;9(5):e01914-18. doi: 10.1128/mBio.01914-18 (PMC6191539; doi:10.1128/mBio.01914-18)
Supplement: FIG S1 [file mbo005184110sf1.pdf]

Supplementary Figure S1

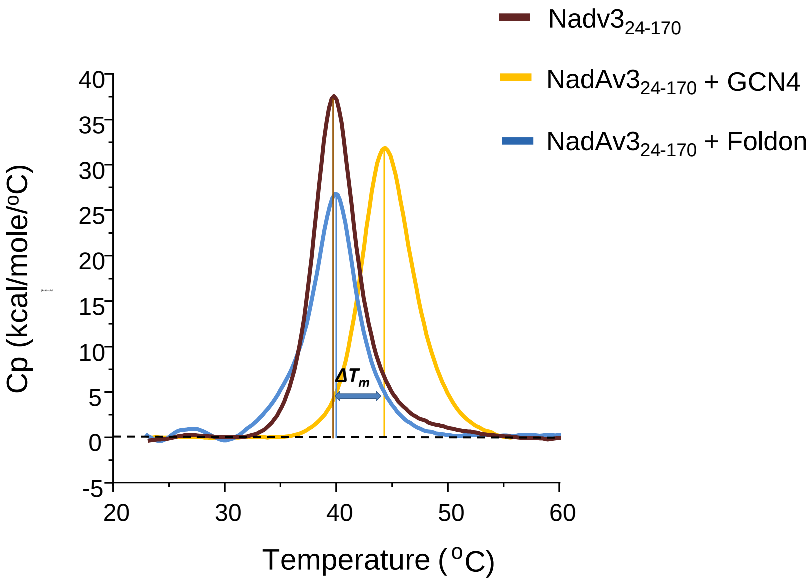

Legend S1. Differential scanning calorimetry (DSC) profiles showing effects of exogenous trimerization motifs on the stability of NadA3 24-170. Genetic fusion of the GCN4 motif to the NadA3 24-170 construct resulted in an increased thermostability ($\Delta\ T_m$ +5 °C), while the Foldon motif did not change the T_m. Experiments were performed in duplicate (n=2); for clarity, one representative curve is shown for each sample.
