# Supplementary material for: NadA3 Structures Reveal Undecad Coiled Coils and LOX1 Binding Regions Competed by Meningococcus B Vaccine-Elicited Human Antibodies
Source: mBio. 2018 Oct 16;9(5):e01914-18. doi: 10.1128/mBio.01914-18 (PMC6191539; doi:10.1128/mBio.01914-18)
Supplement: FIG S2 [file mbo005184110sf2.pdf]

Supplementary Figure S2

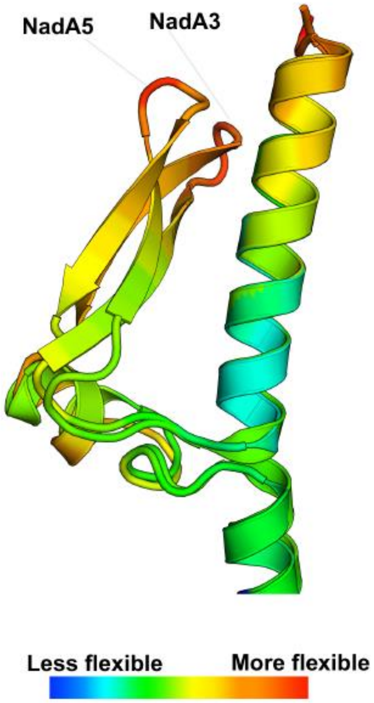

Legend S2. Structural similarities and differences of NadA3 and NadA5 head regions. A zoom into the wing regions, showing the difference in wingtip positions; optimal superposition was obtained aligning residues 28-48 in both structures with the LSQ algorithm. The wingtips are the most flexible regions of both structures, according to B-factor analyses. Crystallographic B-factors represent temperature-dependent vibrations from average atomic positions. Color bar ranges from low B-factor (blue, less flexible) to high B-factor (red, more flexible).
