# Supplementary material for: NadA3 Structures Reveal Undecad Coiled Coils and LOX1 Binding Regions Competed by Meningococcus B Vaccine-Elicited Human Antibodies
Source: mBio. 2018 Oct 16;9(5):e01914-18. doi: 10.1128/mBio.01914-18 (PMC6191539; doi:10.1128/mBio.01914-18)
Supplement: FIG S3 [file mbo005184110sf3.pdf]

Supplementary Figure S3

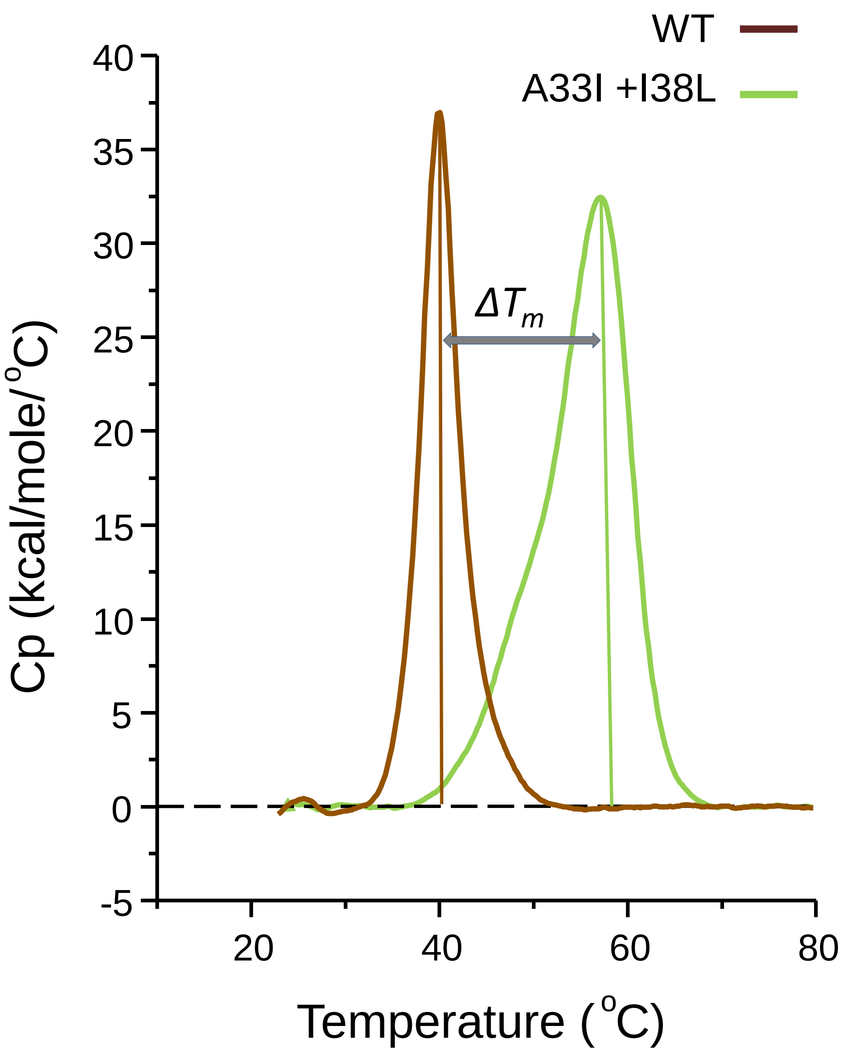

Legend S3. Thermal stabilization of NadA3 by two point mutations. DSC profiles for NadA3 24-170 native protein (dark red) and the most stabilized double-mutant construct: A33I + I38L (orange line) for which the ΔT_m is 17 °C. Experiments were performed in duplicate (n=2); for clarity, one representative curve is shown for each sample.
