# Supplementary material for: NadA3 Structures Reveal Undecad Coiled Coils and LOX1 Binding Regions Competed by Meningococcus B Vaccine-Elicited Human Antibodies
Source: mBio. 2018 Oct 16;9(5):e01914-18. doi: 10.1128/mBio.01914-18 (PMC6191539; doi:10.1128/mBio.01914-18)
Supplement: FIG S4 [file mbo005184110sf4.pdf]

Supplementary Figure S4

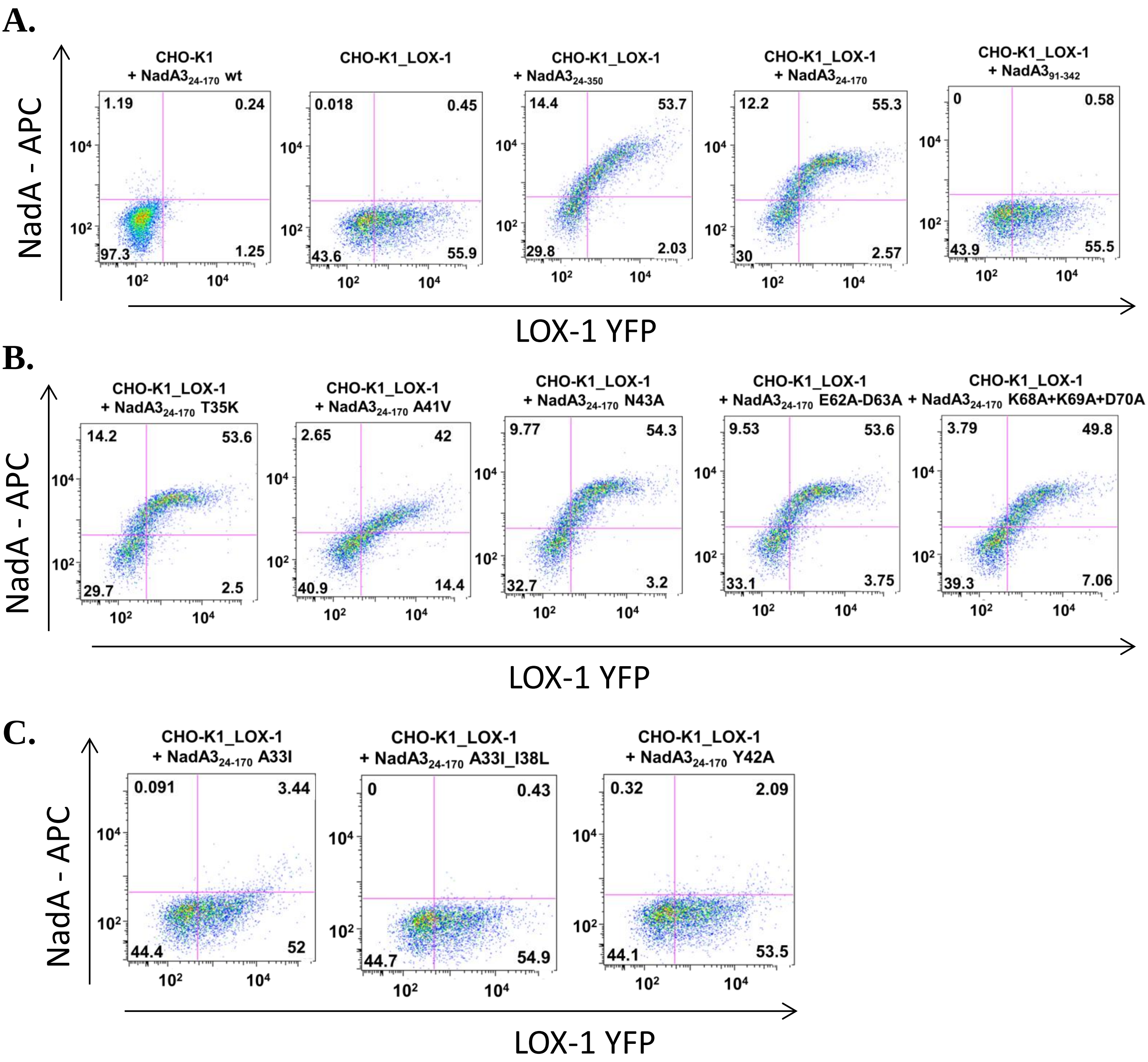

Legend S4. Flow cytometry data for NadA3 24-170 proteins binding to mammalian CHO-K1 cells expressing LOX-1. Representative flow cytometry plots are shown. The gating strategy was designed to separate the cells of interest from large aggregates and debris [initial gate on forward scatter (FSC) versus side scatter (SSC) plot] and doublets/aggregates (standard gates on both FSC-width and SSC-width) (data not shown). Simple gating by quadrants allowed definition of the absolute percentages of cells positive for LOX-1 expression only (LOX-1 transfected cells), cells positive for NadA3 binding only, double negative cells (not-transfected cells) and double positive cells representing cells able to bind recombinant NadA3 via LOX-1. **A)** NadA3 24-170 mediates binding to mammalian cells expressing LOX-1; untransfected CHO-K1 cells are unable to bind NadA (97.3%), the second panel shows cells positive for LOX-1 expression (55.9%), the third and fourth panels show that only LOX-1 transfected cells are able to bind NadA (53.7% for NadA 'full length ectodomain' (24-350) and 55.3% for NadA 24-170), the fifth panel shows that LOX-1 transfected cells (55.5%) are unable to bind the NadA stalk (residues 91-342). **B)** Some mutations in NadA3 24-170 did not affect binding to LOX-1; FACS plots show that LOX-1 transfected cells are able to bind NadA 24-170 mutants (double positive cells: 53.6% for T35K, 42% for A41V, 54.3% for N43A and E62A-D63A and 49.8% for K68A+K69A+D70A). **C)** Some mutations in NadA3 24-170 abolished binding to LOX-1; plots show that LOX-1 transfected cells are unable to bind specific NadA3 24-170 mutants (no double positive cells observed): A33I, A33I+I38L, and Y42A.
