# Supplementary material for: NadA3 Structures Reveal Undecad Coiled Coils and LOX1 Binding Regions Competed by Meningococcus B Vaccine-Elicited Human Antibodies
Source: mBio. 2018 Oct 16;9(5):e01914-18. doi: 10.1128/mBio.01914-18 (PMC6191539; doi:10.1128/mBio.01914-18)
Supplement: FIG S5 [file mbo005184110sf5.pdf]

Supplementary Figure S5

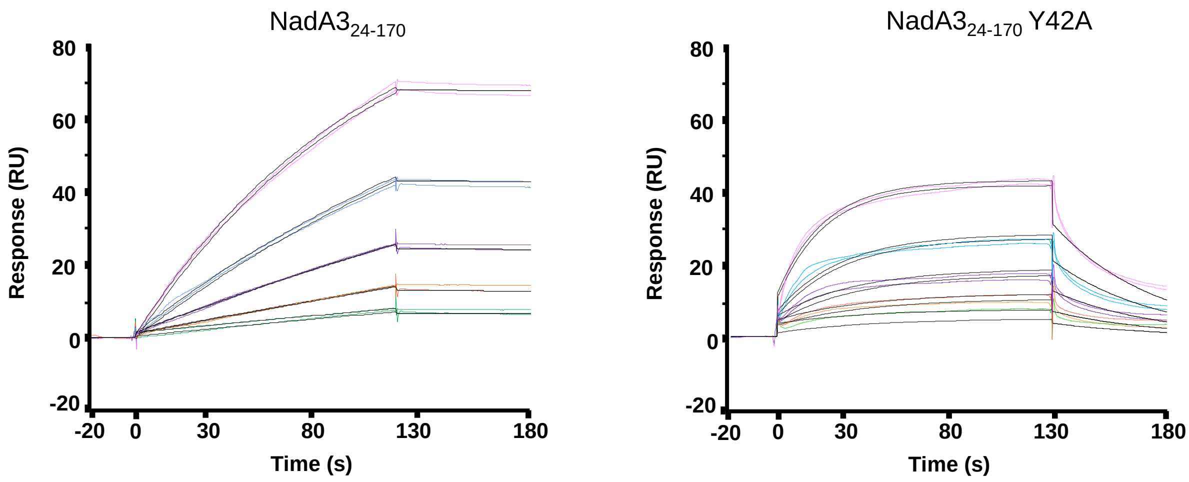

Legend S5. SPR titrations were used to determine the equilibrium dissociation constants (K_D), by injecting multiple increasing concentrations of NadA3 proteins over a sensorchip on which LOX-1 was captured. The Y42A mutant (right, K_D 3.9 ± 0.2 μM) showed approximately 1000-fold weaker binding compared to the native protein (left, K_D 1.8 ± 1 nM). The injection at each concentration was performed in duplicate (n=2), and both experimental curves are shown as colored lines; the black line shows the calculated fit.
