# Supplementary material for: NadA3 Structures Reveal Undecad Coiled Coils and LOX1 Binding Regions Competed by Meningococcus B Vaccine-Elicited Human Antibodies
Source: mBio. 2018 Oct 16;9(5):e01914-18. doi: 10.1128/mBio.01914-18 (PMC6191539; doi:10.1128/mBio.01914-18)
Supplement: FIG S6 [file mbo005184110sf6.pdf]

Supplementary Figure S6

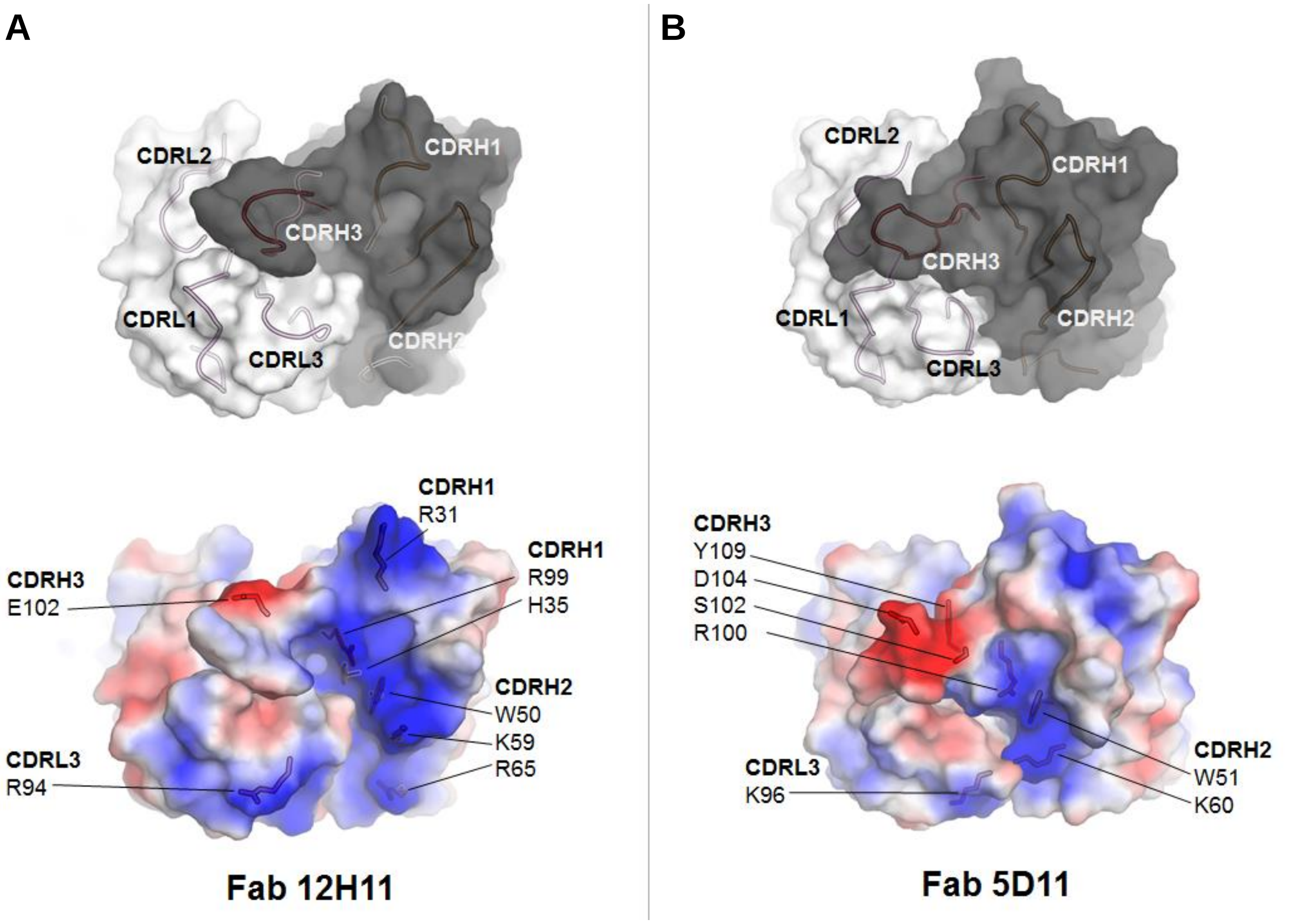

Legend S6: Homology models of the anti-NadA humAbs tested herein, (A) 12H11 and (B) 5D11, both of which expose several postively-charged residues in their paratopes.
