# Supplementary material for: NadA3 Structures Reveal Undecad Coiled Coils and LOX1 Binding Regions Competed by Meningococcus B Vaccine-Elicited Human Antibodies
Source: mBio. 2018 Oct 16;9(5):e01914-18. doi: 10.1128/mBio.01914-18 (PMC6191539; doi:10.1128/mBio.01914-18)
Supplement: FIG S7 [file mbo005184110sf7.pdf]

Supplementary Figure S7

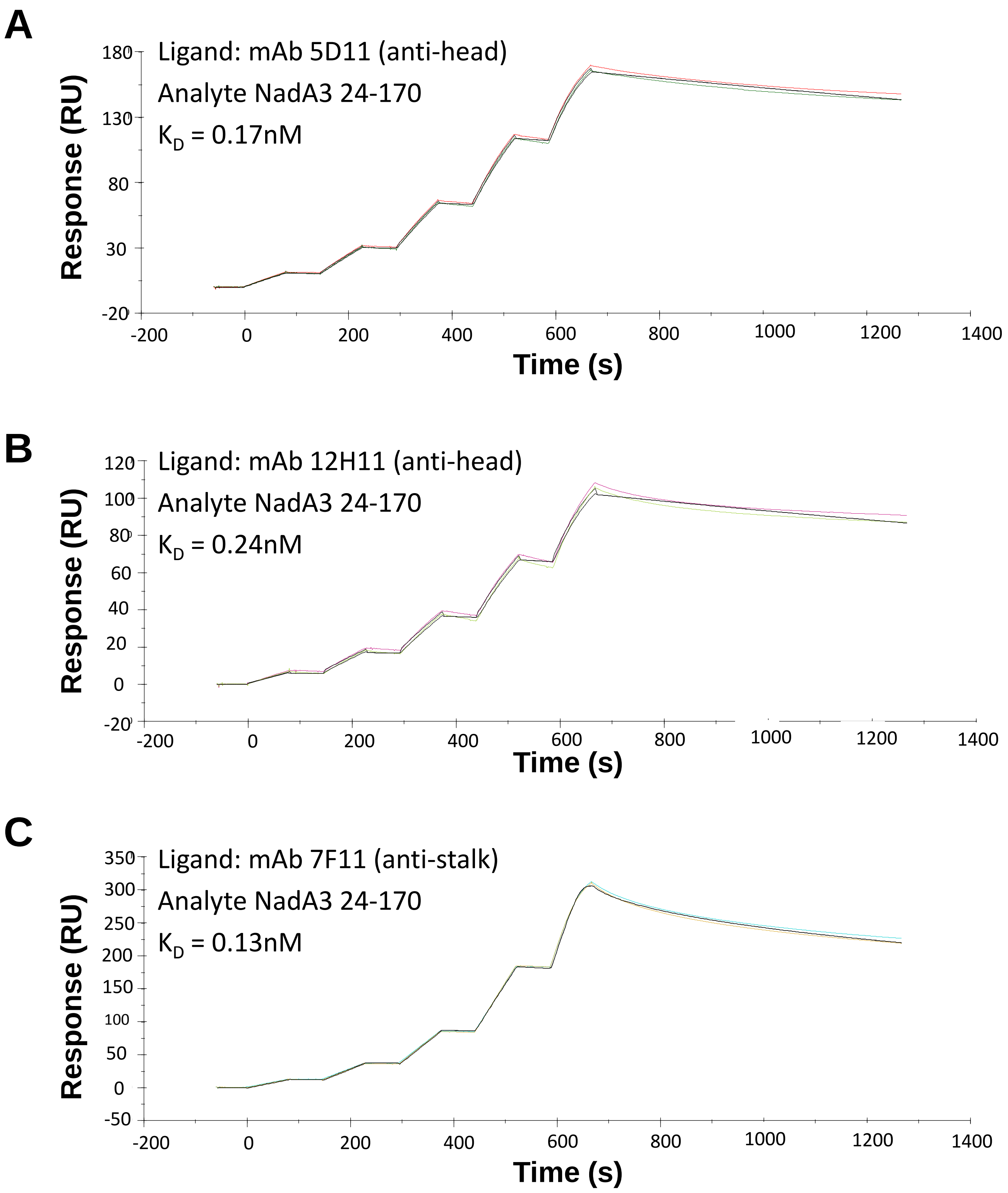

Legend S7: Sensorgrams from SPR single-cycle kinetics experiments with injection of NadA3 over captured human mAbs 5D11 (panel A), 12H11 (panel B) and 7D11 (panel C). Each titration was performed in duplicate (n=2), and both experimental curves are shown as colored lines; the black line shows the calculated fit.
