# Supplementary material for: NadA3 Structures Reveal Undecad Coiled Coils and LOX1 Binding Regions Competed by Meningococcus B Vaccine-Elicited Human Antibodies
Source: mBio. 2018 Oct 16;9(5):e01914-18. doi: 10.1128/mBio.01914-18 (PMC6191539; doi:10.1128/mBio.01914-18)
Supplement: FIG S8 [file mbo005184110sf8.pdf]

Supplementary Figure S8

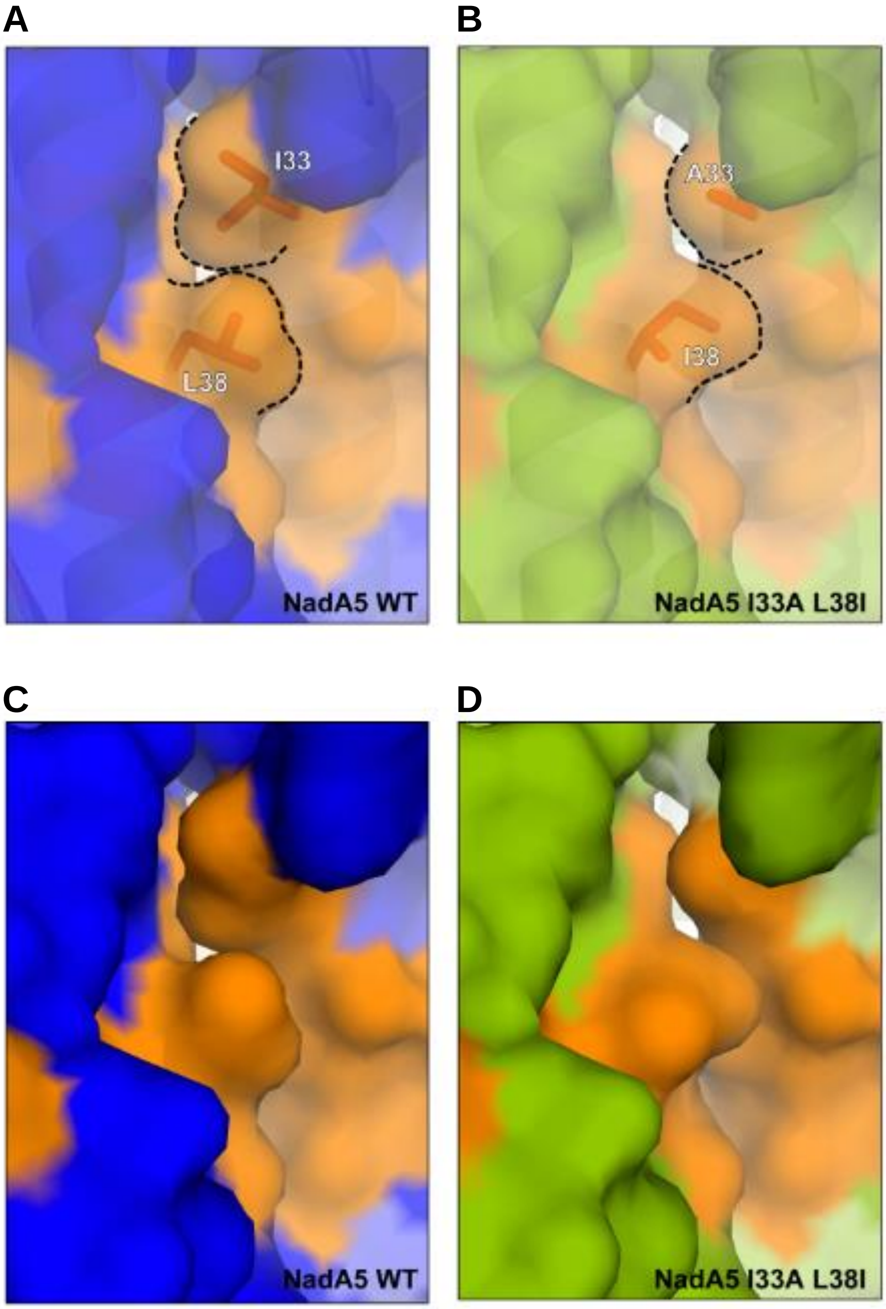

Legend S8: Panels A and B show semi-transparent surface plots of the experimental NadA5 crystal structure (A) and the *in silico* model harboring mutations I33A and L38I (B). Panels C and D show the same molecular views, but with opaque surface rendering. The images serve to illustrate that in wild type NadA5 the residues I33 and L38 make greater Van der Waals' interactions (indicated by black dashed lines) than would be predicted for the NadA5 double mutant, suggesting that the latter would be destabilized compared to wild type.
